# *Alistipes putredinis* Ameliorates Metabolic Dysfunction-Associated Steatotic Liver Disease in Rats via Gut Microbiota Remodeling and Inflammatory Suppression

**DOI:** 10.3390/nu17122013

**Published:** 2025-06-16

**Authors:** Shuwei Zhang, Ruoshi Wang, Ruiqing Zhao, Yao Lu, Mingchao Xu, Xiaoying Lin, Ruiting Lan, Suping Zhang, Huijing Tang, Qianhua Fan, Jing Yang, Liyun Liu, Jianguo Xu

**Affiliations:** 1 National Key Laboratory of Intelligent Tracking and Forecasting for Infectious Diseases, National Institute for Communicable Disease Control and Prevention, Chinese Center for Disease Control and Prevention, Beijing 102206, China; 2 School of Public Health, Nanjing Medical University, Nanjing 211166, China; 3 Department of Epidemiology and Statistics, School of Public Health, Hebei Medical University, Shijiazhuang 050010, China; 4 School of Biotechnology and Biomolecular Sciences, University of New South Wales, Sydney, NSW 2052, Australia; 5 Hebei Key Laboratory of Intractable Pathogens, Shijiazhuang Center for Disease Control and Prevention, Shijiazhuang 050011, China

**Keywords:** MASLD, *Alistipes putredinis*, gut microbiota, inflammation, butyrate, indole derivatives

## Abstract

**Background:** Metabolic dysfunction-associated steatotic liver disease (MASLD) is a highly prevalent chronic liver condition linked to obesity and metabolic imbalance. Alterations in the gut microbiota are increasingly recognized as contributors to its progression. *Alistipes putredinis*, a core member of the human gut microbiota, has been linked with metabolic health, but its functional role in MASLD remains unclear. **Methods:** This study evaluated the potential of *A. putredinis* strain Ap77, isolated from the stool of a healthy adult, to mitigate MASLD-related alterations in a high-fat diet (HFD)-induced rat model. Animals were divided into normal chow (NC), HFD, and HFD plus Ap77 groups and received daily oral gavage of Ap77 or PBS for 8 weeks. **Results:** Ap77 supplementation attenuated the body weight increase associated with high-fat diet consumption. It also reduced hepatic triglyceride levels and fat mass and improved liver histology. Transcriptomic analysis revealed suppression of inflammation-associated pathways. Correspondingly, the concentrations of IL-1β, IL-6, and TNF-α in both the liver and serum were reduced. Ap77 supplementation was associated with an increased abundance of health-associated bacterial genera, such as *Lachnospiraceae UCG_010*, *Akkermansia*, and *Flavonifractor*, as well as elevated serum levels of butyrate, indole-3-propionic acid, and indoleacrylic acid. Notably, correlation analysis revealed that *Lachnospiraceae UCG_010* was positively associated with these metabolites. **Conclusions:** *A. putredinis* Ap77 alleviates hepatic steatosis and inflammation in MASLD, potentially by reshaping gut microbiota and suppressing inflammation-related signaling pathways.

## 1. Introduction

Metabolic dysfunction-associated steatotic liver disease (MASLD) is characterized by hepatic steatosis occurring in individuals with one or more metabolic risk factors and currently affects nearly one in three individuals worldwide [1,2,3,4]. The progression of MASLD involves not only hepatic factors but also extrahepatic mechanisms mediated through the gut–liver axis, in which dietary habits, host genetics, and intestinal microbial alterations jointly contribute to disease development [5,6]. Patients with MASLD have been reported to show a higher abundance of Proteobacteria and Firmicutes and a lower abundance of Bacteroidetes [7,8,9]. Gut microbiota modulation is considered a potential therapeutic approach for the management of MASLD [10]. Among these strategies, probiotics and prebiotics have shown promise in ameliorating hepatic steatosis and inflammation by modulating gut microbiota composition, enhancing intestinal barrier integrity, and regulating host metabolic and immune pathways [11].

*Alistipes putredinis* is predominantly found in the gut of healthy individuals and has been identified as a core species of the human gut microbiota [12]. It belongs to the phylum Bacteroidota, family *Rikenellaceae*, and genus *Alistipes* [13,14]. Population cohort studies have confirmed that *A. putredinis* can modulate the relationship between physical activity and body weight [15,16]. Moreover, its abundance has been consistently reduced in patients with MASLD [17]. This reduction shows a negative correlation with liver fibrosis progression, with *A. putredinis* levels declining as fibrosis severity increases [18]. Emerging evidence suggests that *A. putredinis* may contribute to metabolic health by supporting gut barrier function and modulating immune responses [19,20]. Notably, *A. putredinis* has also exhibited anti-inflammatory properties and was shown to alleviate inflammatory bowel disease in murine models via IL-10 induction and TLR2-mediated signaling [21].

Despite increasing evidence supporting the role of *A. putredinis* in metabolic health, its specific involvement in MASLD has not been experimentally validated. Given that MASLD is a multifactorial disease involving lipid metabolism disorders, insulin resistance, chronic inflammation, and gut–liver axis disruption, further investigation into host–microbiota interactions is needed. High-fat diet (HFD)-induced rat models effectively replicate key pathological features of MASLD and are widely used for mechanistic studies [22]. In this study, we employed *A. putredinis* strain Ap77, a fecal isolate from a healthy Chinese adult that was previously obtained and preserved by our research team [23], to examine its regulatory effects in an MASLD rat model.

## 2. Materials and Methods

### 2.1. Bacterial Culture

*A. putredinis* Ap77 was cultured anaerobically at 37 °C for 48 h on brain heart infusion (BHI) agar (OXOID, Thermo Fisher Scientific, Waltham, MA, USA) containing 5% defibrinated sheep blood.

### 2.2. Experimental Animals and Model Induction

All animal procedures were performed following previously described protocols. [24]. Briefly, Male Wistar rats (180 ± 20 g) of specific-pathogen-free (SPF) grade were housed under SPF conditions with controlled temperature and humidity and a 12 h/12 h light/dark cycle and fed and watered without restriction. After one week of acclimation, 24 rats were randomly divided into three groups: NC (normal chow, D12450J; 10% fat), HFD (high-fat diet, D12492; 60% fat), and Ap77 (HFD supplemented with *A. putredinis* Ap77). Both diets were purchased from Research Diets Inc. (New Brunswick, NJ, USA). Then for 8 weeks, rats in the Ap77 group were orally gavaged once daily with 2.0 × 10^9^ CFU of *A. putredinis* Ap77 suspended in 2 mL PBS, while rats in the NC and HFD groups received 2 mL PBS alone. Body weight and food consumption were recorded on a weekly basis, and the food efficiency ratio (FER) was subsequently calculated. At the end of the study, all rats were deeply anesthetized with sodium pentobarbital (50 mg/kg, intraperitoneally) before sample collection and were humanely euthanized in accordance with institutional ethical guidelines, with pain and distress being minimized. Samples were collected for subsequent biochemical and histological analyses, including serum enzymes, lipid profiles, hepatic triglycerides, and inflammatory cytokines, which were evaluated using standard biochemical analyzers and ELISA kits (Dogesce, Beijing, China). Hematoxylin and eosin (H&E) staining was performed on liver and adipose tissues, and liver sections were evaluated according to the nonalcoholic fatty liver disease (NAFLD) Activity Scoring system [25]. No expected or unexpected adverse events were observed during the experimental period. No animals were excluded during the study. Confounding factors such as housing, handling, and feeding conditions were strictly controlled under the SPF environment to minimize bias. This study was approved by the Animal Welfare and Ethics Committee of the Chinese CDC (Approval No. 2022-036).

### 2.3. Liver Transcriptome Analysis

Total RNA from liver tissues was isolated using TRIzol reagents (Invitrogen, Carlsbad, CA, USA) following standard protocols as previously described [26]. Library preparation and high-throughput RNA sequencing were carried out by Biomarker Technologies (Beijing, China). Differential expression was evaluated using DESeq2, and genes were considered significantly regulated when |log_2_FC| was greater than 1 and the adjusted *p*-value was smaller than 0.05. Gene set enrichment analysis (GSEA) was performed using the ranked gene list derived from differential expression analysis. Kyoto Encyclopedia of Genes and Genomes (KEGG) pathway gene sets served as the reference database, and pathways with an adjusted *p* < 0.05 were regarded as significantly enriched. RNA-seq datasets have been publicly archived in the NCBI SRA (PRJNA1253867).

### 2.4. Gut Microbiota Composition Analysis

Total genomic DNA was extracted from fecal samples using the TGuide S96 Magnetic Stool DNA Kit (Tiangen Biotech, Beijing, China), following the manufacturer’s protocol. The V3–V4 hypervariable regions of the bacterial 16S rRNA gene were amplified using primers 338F (5′-ACTCCTACGGGAGGCAGCAG-3′) and 806R (5′-GGACTACHVGGGTWTCTAAT-3′), both tailed with Illumina adapter sequences. PCR reactions (10 μL) included 5–50 ng of DNA template, 0.3 μL of each primer (10 μM), 5 μL of KOD FX Neo Buffer, 2 μL of dNTPs (2 mM each), 0.2 μL of KOD FX Neo polymerase (Toyobo, Osaka, Japan), and ddH_2_O to volume. The amplification protocol was as follows: 95 °C for 5 min; 20 cycles of 95 °C for 30 s, 50 °C for 30 s, and 72 °C for 40 s; and a final extension at 72 °C for 7 min. Amplicons were purified using an Omega DNA purification kit (Omega Bio-Tek, Norcross, GA, USA) and quantified with a Qsep-400 system (BiOptic Inc., New Taipei City, Taiwan, China). Paired-end sequencing (2 × 250 bp) was performed on the Illumina NovaSeq 6000 platform (Beijing Biomarker Technologies Co., Ltd., Beijing, China). Bioinformatic analysis was conducted using QIIME2 (v2020.6) [27], including ASV clustering with DADA2 and taxonomic classification based on the SILVA database (release 138.1) with a 70% confidence threshold [28]. Alpha and beta diversity indices were calculated within the same framework. Principal Coordinate Analysis (PCoA) based on unweighted UniFrac metrics was used to visualize group differences in microbial community composition, and statistical significance was assessed by permutational multivariate analysis of variance (PERMANOVA) [29]. To identify discriminatory taxa among groups, LEfSe (LDA score > 4) was used to detect significantly enriched taxa across groups. The 16S rRNA amplicon sequencing data have been deposited in the NCBI Sequence Read Archive (SRA) (PRJNA1250185).

### 2.5. Serum Metabolites Analysis

Broad-targeted metabolomics was conducted to analyze serum metabolites. Serum samples were mixed with a pre-cooled methanol/acetonitrile/water solution (2:2:1, *v*/*v*/*v*) containing a mixture of stable-isotope-labeled internal standards, vortexed, and centrifuged at 14,000× *g* for 20 min at 4 °C. The internal standards included Malic Acid-d3, Adipic acid-d10, Melatonin-d4, 8-isoPGF2α-d4, GUDCA-d4, SCFA-d5, CA-d5, TLCA-d4, GLCNA-d4, DHA-d5, OA-d5, Creatinine-d3, Tyrosine-d4, Carnitine-D9C3, threonine-D5C1, Histidine-15N3, Serine-13C3-15N, Glutamate-d5, Arginine-d7, Isoleucine-d10, Nicotinic acid-d4, Glycine-15N, UCDA-d4, CA2-d4, Proline-d7, Valine-d8, Lactic acid-d3, Citric acid-d4, Fumaric acid-d4, and Succinic acid-d4. The resulting supernatant was analyzed using multiple reaction monitoring (MRM) LC–MS/MS (Agilent, Santa Clara, CA, USA). Metabolites were identified by retention time and quantified using external standards. Data were normalized (median scaling) and autoscaled prior to analysis. The ropls R package was used to conduct Partial Least Squares Discriminant Analysis (PLS-DA), and metabolites showing |log_2_FC| > 1, Variable Importance in Projection (VIP) > 1, and *p* < 0.05 were considered significantly different. One-way ANOVA was used for further validation. Short-chain fatty acids (SCFAs) in serum were quantified using GC–MS (Shimadzu, Kyoto, Japan) equipped with an HP-FFAP capillary column. Lyophilized serum was mixed with sulfuric acid and methyl tert-butyl ether containing internal standards 2-methylglutaric acid (25 mg/L), then vortexed, sonicated, and centrifuged. The resulting supernatant was analyzed under standard GC–MS conditions. SCFAs were identified by retention time and quantified using calibration curves.

### 2.6. Statistical Analysis

Normality and homogeneity of variance were evaluated for each dataset prior to statistical analysis. For data meeting these assumptions, one-way or two-way ANOVA was applied as appropriate. Differences in body weight trajectories across groups were tested using two-way ANOVA. When variance homogeneity was not satisfied, Welch’s ANOVA or Brown–Forsythe ANOVA was employed. Non-normally distributed data were analyzed using nonparametric tests. Multiple comparisons were corrected using the Benjamini–Hochberg method to control the false discovery rate (FDR), with *p* < 0.05 considered statistically significant. Associations between microbial taxa and serum metabolites were examined via Spearman’s rank correlation analysis. Data was shown as the mean with standard deviation (SD).

## 3. Results

### 3.1. Ap77 Influences Physiological Parameters in MASLD Rats

**Figure 1:**
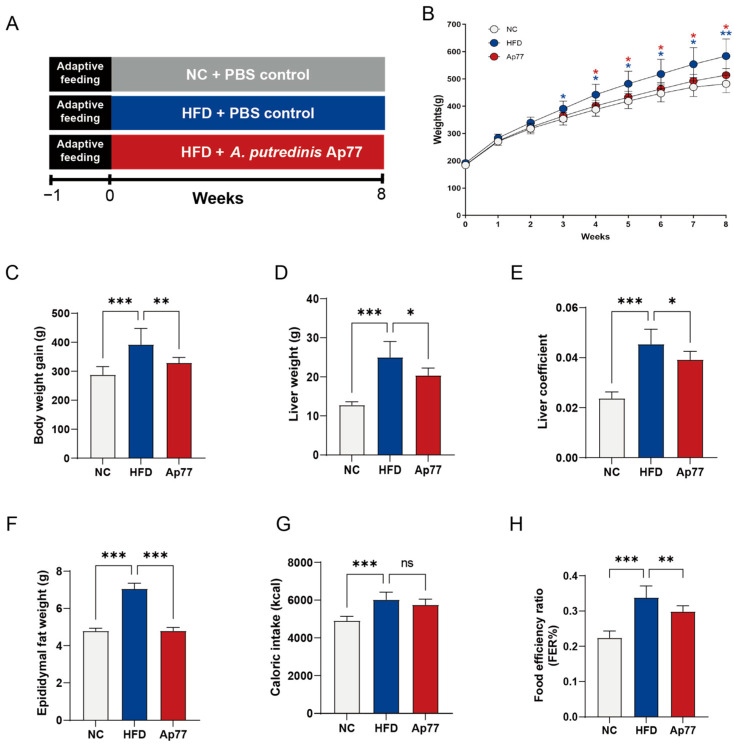
Ap77 administration alters weight gain, consumption, and tissue mass. (**A**) Overview of the experimental setup. (**B**) Weekly changes in body weight. (**C**) Total body weight gain. (**D**) Liver weight. (**E**) Liver coefficient. (**F**) Epididymal fat weight. (**G**) Caloric intake. (**H**) Food efficiency ratio (FER). Data are presented as mean ± SD (*n* = 8 per group). * *p* < 0.05; ** *p* < 0.01; *** *p* < 0.001; ns, not significant.

An MASLD rat model was established to evaluate the protective effect of Ap77 (Figure 1A). The body weight of the HFD group was significantly increased compared with the NC group, while Ap77 administration effectively mitigated this effect (*p* < 0.05; Figure 1B,C). While HFD-fed rats exhibited increased liver weight, epididymal fat mass, and liver index values relative to the NC group, these parameters were effectively reduced by Ap77 intervention (*p* < 0.05; Figure 1D–F). No significant difference in caloric intake was observed between the HFD and Ap77 groups (*p* > 0.05; Figure 1G). The food efficiency ratio was elevated in the HFD group and markedly reduced by Ap77 treatment (*p* < 0.001; Figure 1H).

### 3.2. Ap77 Ameliorates Liver Function Impairment in MASLD Rats

**Figure 2:**
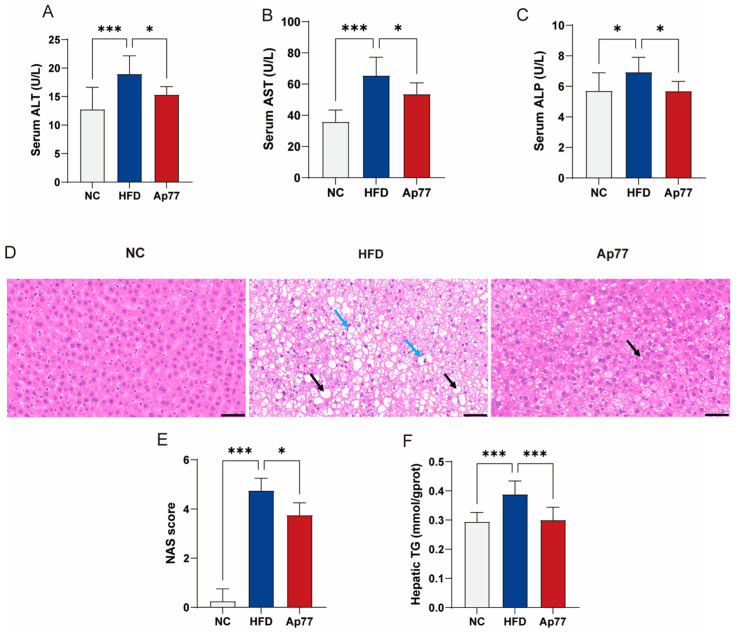
Effects of Ap77 on liver function in MASLD rats. (**A**) Serum ALT levels. (**B**) Serum AST levels. (**C**) Serum ALP levels. (**D**) H&E staining of liver sections; black arrows indicate steatosis and blue arrows indicate ballooning (scale bar, 50 μm). (**E**) NAS. (**F**) Hepatic TG content. Data are presented as mean ± SD (*n* = 8 per group). * *p* < 0.05, *** *p* < 0.001.

Serum levels of alanine Aminotransferase (ALT), aspartate Aminotransferase (AST), and alkaline Phosphatase (ALP) were markedly increased in the HFD group relative to the NC group but were reduced by Ap77 supplementation (*p* < 0.05; Figure 2A–C). H&E staining revealed that hepatocytes in the NC group exhibited a uniform shape and size. Hepatic cords were well organized, and no lipid droplets were observed in liver cells, indicating a normal hepatic lobular structure. In contrast, hepatic tissues in the HFD group displayed marked hepatocyte steatosis (black arrows) and ballooning degeneration (blue arrows). The Ap77 group exhibited fewer steatotic hepatocytes and reduced hepatic lipid accumulation compared to the HFD group (Figure 2D). The HFD group showed a marked elevation in NAFLD activity score (NAS) compared to the NC group, whereas Ap77 supplementation led to a significant reduction (*p* < 0.05; Figure 2E). The hepatic triglycerides (TG) level, which was elevated in the HFD group, was reduced in the Ap77 group (*p* < 0.001; Figure 2F).

### 3.3. Ap77 Modulates Lipid Profile and Adipose Tissue Morphology in MASLD Rats

**Figure 3:**
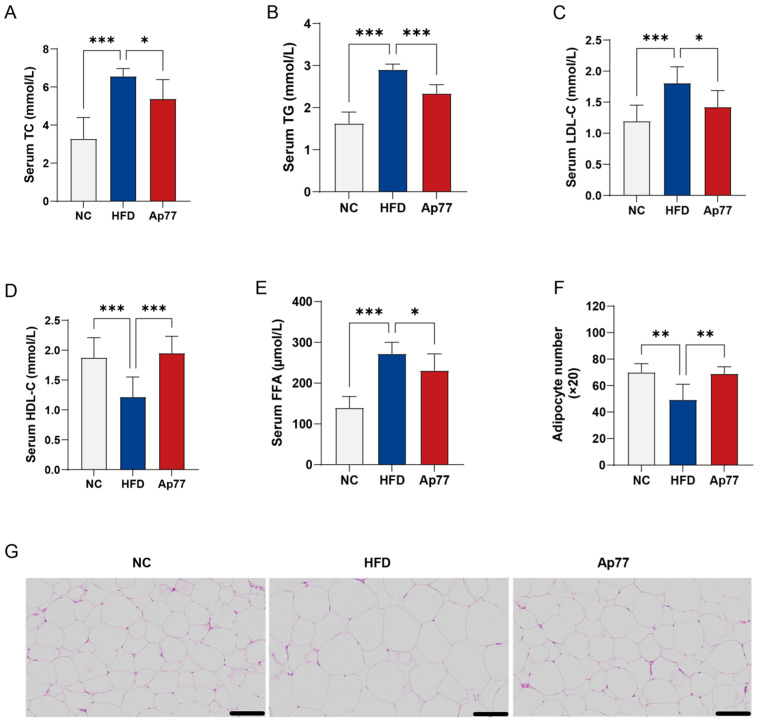
Serum lipid regulation and adipose morphology by Ap77. (**A**) Serum TC levels. (**B**) Serum TG levels. (**C**) Serum LDL-C levels. (**D**) Serum HDL-C levels. (**E**) Serum FFA levels. (**F**) H&E-stained sections of epididymal adipose tissue; scale = 100 μm. (**G**) Adipocyte number. Data are presented as mean ± SD (*n* = 8 per group). * *p* < 0.05, ** *p* < 0.01, *** *p* < 0.001.

HFD feeding led to significant increases in serum total cholesterol (TC), TG, Low-density lipoprotein cholesterol (LDL-C), and free fatty acids (FFA) levels compared to the NC group, while Ap77 administration markedly reduced these elevated lipid parameters (*p* < 0.05; Figure 3A–C,E). High-density lipoprotein cholesterol (HDL-C) was significantly lower in the HFD group relative to the NC group, whereas Ap77 restored it to near-normal levels (*p* < 0.001; Figure 3D). In addition, under the same microscopic field, there were fewer adipocytes in the HFD group than in the NC group, suggesting adipocyte hypertrophy. This change was reversed by Ap77 intervention (*p* < 0.05; Figure 3F,G).

### 3.4. Ap77 Suppresses Inflammation-Related Pathways

**Figure 4:**
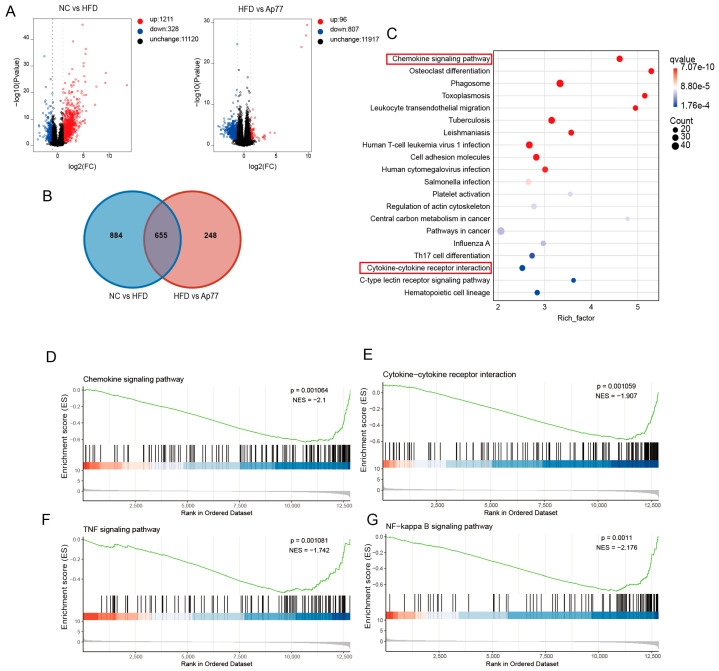
Transcriptomic analysis of liver tissues. (**A**) Volcano plot of the DEGs’ distribution. DEGs: red = upregulated; blue = downregulated. (**B**) Venn diagram showing overlapping DEGs across groups. (**C**) Bubble plot of KEGG-enriched pathways for DEGs shared among the three groups. Red boxes indicate inflammation-related pathways identified by KEGG analysis. (**D**–**G**) GSEA results for inflammation-related pathways. The green curve represents the enrichment score (ES); red indicates upregulated genes and blue indicates downregulated genes.

Transcriptome profiling of liver tissues identified 1539 differentially expressed genes (DEGs) in the HFD group relative to the NC group, comprising 1211 upregulated and 328 downregulated genes. In comparison, 903 DEGs were detected in the Ap77 group, including 96 upregulated and 807 downregulated genes (Figure 4A). KEGG pathway enrichment analysis revealed that inflammation-related pathways were prominently represented among the 655 DEGs shared by all three groups (Figure 4B,C). Consistently, GSEA showed that cytokine–cytokine receptor interaction (ko04060), chemokine signaling (ko04062), TNF signaling (ko04668), and NF-κB signaling (ko04064) in the HFD group were significantly increased compared to the NC group (Figure S1A–D) but were notably downregulated following Ap77 intervention (Figure 4D–G). The expression of all DEGs is shown in a heatmap (Figure S1E).

### 3.5. Ap77 Attenuates Inflammatory Responses in MASLD Rats

**Figure 5:**
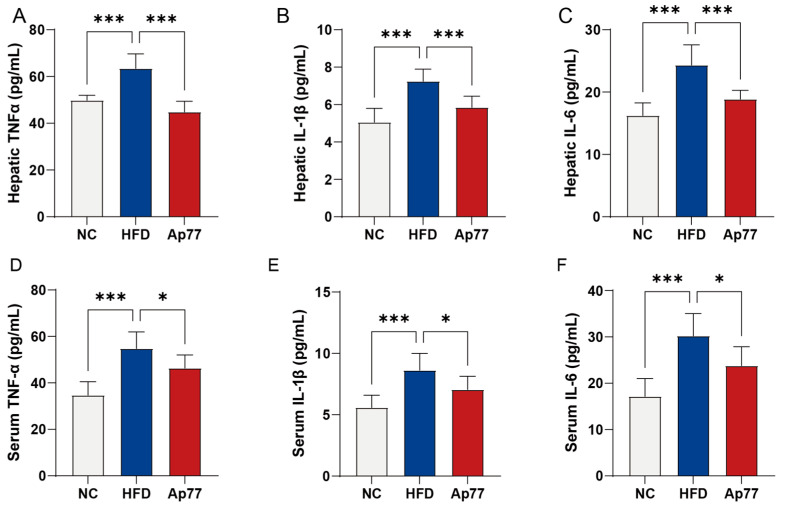
Changes in hepatic and serum cytokines following Ap77 administration. (**A**–**C**) Hepatic levels of TNF-α, IL-1β, and IL-6. (**D**–**F**) Circulating concentrations of TNF-α, IL-1β, and IL-6 in serum. Data are presented as mean ± SD (*n* = 8 per group). * *p* < 0.05, *** *p* < 0.001.

Hepatic and serum levels of TNF-α, IL-1β, and IL-6 were quantified. The levels of these cytokines were markedly higher in the HFD group than in the NC group. Notably, Ap77 treatment markedly lowered their concentrations relative to the HFD group (*p* < 0.05; Figure 5A–F).

### 3.6. Gut Microbiota Remodeling by Ap77 in MASLD Rats

**Figure 6:**
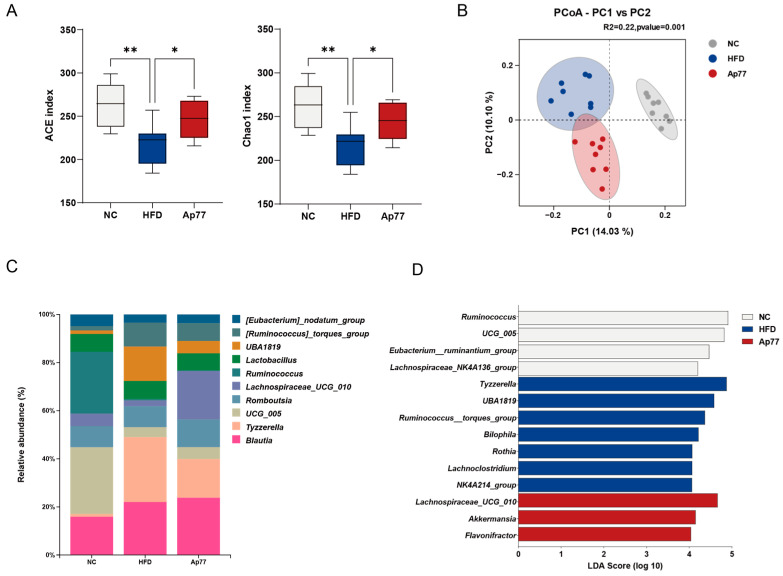
Effects of Ap77 on gut microbiota. (**A**) α-diversity assessed by ACE and Chao1 indices. (**B**) β-diversity visualized by PCoA. (**C**) Genus-level composition of fecal microbiota. (**D**) Differentially abundant genera identified by LEfSe analysis. Data are presented as mean ± SD (*n* = 8 per group). * *p* < 0.05; ** *p* < 0.01.

Sequencing depth and quality control met analysis requirements (Figure S2A). For α-diversity, the abundance-based coverage estimator (ACE) and Chao1 indices were decreased in the HFD group relative to the NC group but increased in the Ap77 group (*p* < 0.05; Figure 6A). PCoA revealed distinct clustering patterns in β-diversity among the three groups (Figure 6B). At the phylum level, Firmicutes were predominant across all groups (Figure S2B). The Firmicutes-to-Bacteroidota ratio (F/B) was increased in the HFD group compared to the NC group and was partially reversed in the Ap77 group (Figure S2C). At the genus level, *UCG_005*, *Ruminococcus*, and *Blautia* were enriched in the NC group; *Tyzzerella*, *Blautia*, and *UBA1819* were more abundant in the HFD group; and *Blautia*, *Lachnospiraceae UCG_010*, and *Tyzzerella* were dominant in the Ap77 group (Figure 6C). LEfSe identified *Ruminococcus* and *UCG_005* as taxa markedly enriched in the NC group. The HFD group was characterized by the enrichment of genera including *Tyzzerella* and *UBA1819*, while the Ap77 group showed significantly higher abundances of *Lachnospiraceae UCG_010*, *Akkermansia*, and *Flavonifractor* (Figure 6D). In addition, Metastats analysis showed a statistically significant increase in the relative abundance of the *Alistipes* genus in the Ap77 group compared to the HFD group (Figure S2D).

### 3.7. Modulation of Serum Metabolites by Ap77 in MASLD Rats

**Figure 7:**
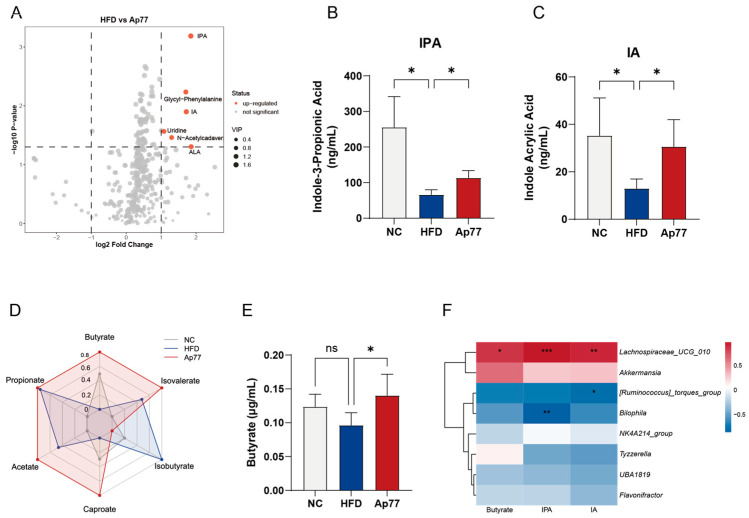
Serum metabolite alterations induced by Ap77 in MASLD rats. (**A**) Volcano plot of HFD vs. Ap77 metabolites. The dashed lines indicate the thresholds for log2 fold change and p-value. (**B**,**C**) Serum IPA and IA levels. (**D**) Radar chart of average SCFA profiles. (**E**) Serum butyrate levels. (**F**) Spearman correlation between key genera and metabolites. Colors denote correlation direction: red for positive and blue for negative. Asterisks denote significant correlations. Data are presented as mean ± SD (*n* = 4 per group). * *p* < 0.05; ** *p* < 0.01; *** *p* < 0.001; ns, not significant.

A volcano plot was used to visualize metabolic differences between the HFD and Ap77 groups, revealing six metabolites that were significantly elevated following Ap77 treatment (Figure 7A). The NC vs. HFD comparison revealed significant modulation of 19 metabolites, among which 5 were upregulated and 14 were downregulated (Figure S3A). One-way ANOVA applied to raw concentration data further verified that levels of indole-3-propionic acid (IPA) and indoleacrylic acid (IA) were significantly diminished in the HFD group but notably restored upon Ap77 treatment (Figure 7B,C). Conversely, no statistically significant changes were observed in the levels of α-linolenic acid (ALA), glycyl-phenylalanine (Gly-Phe), N-acetylcadaverine, and uridine across the experimental groups (Figure S3B–E).

The radar chart revealed a general reduction in multiple SCFAs (e.g., butyrate, propionate, and caproate) in the HFD group compared with the NC group. Ap77 supplementation showed an overall increasing trend in SCFA levels (Figure 7D). Quantitative analysis confirmed that compared to the NC group, the butyrate level was significantly lower in the HFD group; however, Ap77 treatment increased butyrate levels (*p* < 0.05; Figure 7E). Concentrations of acetate, propionate, caproate, isobutyrate, and isovalerate did not differ significantly among the groups (Figure S3F–J).

As shown in Figure 7F, several key metabolites exhibited significant correlations with specific gut microbial genera. Notably, *Lachnospiraceae_UCG_010*, enriched in the Ap77 group, showed significant positive correlations with butyrate, IPA, and IA (*p* < 0.05). In contrast, both the *Ruminococcus_torques* group and *Bilophila*, which were enriched in the HFD group, exhibited negative correlations: the *Ruminococcus_torques* group was negatively correlated with IA (*p* < 0.05), while *Bilophila* showed a significant negative correlation with IPA (*p* < 0.01).

## 4. Discussion

*Alistipes* is a relatively new genus within *Bacteroidetes* and has attracted increasing attention in recent years due to its potential roles in host metabolism and immunity [13]. In this study, we demonstrated that *A. putredinis* strain Ap77 effectively ameliorates liver injury, lipid metabolic disturbances, and both systemic and hepatic inflammation in HFD-induced MASLD rats. These improvements are potentially driven by the changes in the gut microbiota and its metabolites.

In this study, *A. putredinis* Ap77 supplementation significantly attenuated body weight gain and reduced liver and epididymal fat mass in rats fed a high-fat diet. Notably, both the HFD and Ap77 groups were subjected to the same high-fat dietary regimen, indicating that the observed improvements in weight were not due to differences in caloric intake. Histological analysis of epididymal adipose tissue further supported this observation: under identical magnification, the number of adipocytes per field was markedly higher in the Ap77 group than in the HFD group, suggesting smaller adipocyte size and reduced hypertrophy. These morphological differences indicate that Ap77 may prevent adipocyte hypertrophy and limit visceral fat expansion. Collectively, these findings demonstrate that Ap77 effectively modulates host weight gain and lipid storage under obesogenic conditions. This aligns with previous evidence showing that specific probiotic combinations targeting multiple metabolic pathways can alleviate diet-induced adiposity, reduce fat accumulation, and regulate host energy metabolism in high-fat diet models [30].

Chronic low-grade inflammation is a well-established hallmark of MASLD and plays a central role in disease progression [31,32,33]. Hepatocytes exposed to excessive lipid accumulation release pro-inflammatory cytokines such as IL-1β, IL-6, and TNF-α, which promote hepatic injury, immune cell infiltration, and amplification of the inflammatory response [34]. Among these pathways, the TNF signaling cascade—particularly the key upstream mediator TNFα—not only induces hepatocyte apoptosis but also activates downstream targets such as the NF-κB pathway, thereby enhancing pro-inflammatory cytokine expression and intensifying immune responses [35]. These mediators are critically involved in both the “first hit” and “second hit” stages of MASLD, contributing to the transition from simple steatosis to steatohepatitis, fibrosis, and ultimately cirrhosis [36]. In this study, Ap77 intervention markedly reduced hepatic triglyceride accumulation, thereby alleviating the lipid burden that drives inflammation. Transcriptomic sequencing further revealed that Ap77 downregulated several major inflammation-related pathways. These pathways converge on the regulation of pro-inflammatory cytokines, consistent with our observation that the IL-1β, IL-6, and TNF-α levels were significantly reduced in both the liver and serum through Ap77 intervention. This dual effect underscores the link between hepatic and systemic inflammation in MASLD. Hepatic inflammation not only contributes to local tissue damage but also facilitates the spillover of cytokines into the circulation, thereby amplifying systemic immune activation. Conversely, circulating inflammatory mediators may further exacerbate hepatic injury, forming a vicious cycle that sustains disease progression. This self-perpetuating loop may be further intensified by bidirectional crosstalk between innate and adaptive immunity, as recently proposed by Sawada et al. [37]. In this context, the observed reductions in hepatic and serum cytokines following Ap77 treatment suggest a disruption of this immuno-inflammatory feedback loop. Collectively, these findings confirm the attenuation of inflammation at both local and systemic levels and support the role of inflammation as a central therapeutic target in MASLD management. Taken together, these findings suggest that Ap77 alleviates MASLD, at least in part, by disrupting the lipid-induced activation of inflammation-related signaling cascades, thereby supporting the concept of inflammation as a critical therapeutic target in MASLD management [38].

There is growing evidence suggesting a strong link between MASLD and gut microbiota changes [39,40,41]. Previous studies have indicated that MASLD-related dysbiosis of gut microbiota causes disruption of the intestinal barrier, resulting in the movement of gut microbial metabolites such as lipopolysaccharide (LPS) from the intestine to the liver through blood circulation, leading to systemic inflammation and liver injury [42]. In our study, Ap77 administration effectively mitigated HFD-induced dysbiosis in MASLD rats, as demonstrated by the restoration of α-diversity indices and the partial reversal of significant shifts in β-diversity. Moreover, an increased Firmicutes-to-Bacteroidota (F:B) ratio has been widely associated with gut microbial dysbiosis in MASLD and obesity and is considered a hallmark of hepatic steatosis and metabolic imbalance [43]. In our study, Ap77 treatment significantly reduced the F/B ratio compared to the HFD group, suggesting that Ap77 may help alleviate gut microbial disturbances associated with MASLD. Similar to our observation, it has been reported that *Tyzzerella*, *UBA1819*, and *Ruminococcus torques* group were significantly enriched in HFD mice [44,45]. These taxa were likewise elevated in the HFD group in our study, suggesting reproducible patterns of microbial dysbiosis associated with MASLD. Notably, Ap77 treatment significantly increased the abundance of *Lachnospiraceae* UCG_010, *Akkermansia*, and *Flavonifractor*, consistent with the study of *Yang* et al., in which grape powder intake was shown to increase microbial diversity and promote the growth of beneficial taxa [46]. Additionally, *Akkermansia muciniphila* is regarded as a promising “next-generation beneficial microbe” due to its ability to regulate lipid metabolism and reduce inflammation, thereby alleviating MASLD [47,48]. Furthermore, *Flavonifractor plautii* was reported to attenuate inflammatory responses in obese adipose tissue [49]. Moreover, the observed increase in *Alistipes* abundance following Ap77 treatment may further contribute to its anti-inflammatory and metabolic benefits, as members of this genus have been associated with improved gut barrier function and reduced hepatic inflammation [13,50].

In many cases, the positive effects of gut microorganisms are not exerted directly but occur through bioactive microbial metabolites [5,51]. IPA and IA, products of gut-microbiota-dependent tryptophan metabolism, are classified as indole derivatives. These microbial metabolites exert immunomodulatory effects and contribute to the regulation of host inflammatory pathways [52]. Supplementation with IPA has been shown to attenuate NF-κB pathway activity through a reduction in endotoxin levels and suppression of macrophage activation [53]. IA exhibits anti-inflammatory activity by suppressing IL-6 and IL-1β production in LPS-treated peripheral blood mononuclear cells [54]. Butyrate has also attracted attention as a potent microbial metabolite with therapeutic potential. It has been reported that butyrate alleviates steatohepatitis by limiting inflammatory macrophage activity [55] and may have beneficial effects on MASLD through inhibition of NF-κB nuclear translocation and modulation of inflammatory signaling [56]. In the present study, significantly elevated serum levels of butyrate, IPA, and IA were observed following Ap77 intervention. These changes were accompanied by reduced hepatic and serum inflammatory markers, suggesting an anti-inflammatory mechanism mediated by altered microbial metabolites. Previous studies have shown that *Lachnospiraceae_UCG_010*, *Akkermansia*, and *Flavonifractor* are major SCFA-producing genera, with butyrate in particular being known for its anti-inflammatory effects [57,58]. Consistent with these findings, our study observed a significant enrichment of these genera in the Ap77 group, suggesting that Ap77 may enhance SCFA production and contribute to inflammation resolution via modulation of the gut microbiota. Correlation analysis further revealed that *Lachnospiraceae_UCG_010* was positively associated with butyrate, IPA, and IA. Conversely, *Ruminococcus_torques_group* and *Bilophila*, both enriched in the HFD group, showed negative correlations with IA and IPA, respectively. These findings are consistent with previous evidence suggesting that microbial metabolites such as indole derivatives and SCFAs, produced by specific gut bacteria, contribute to the amelioration of metabolic and inflammatory disturbances in MASLD models [53,59,60]. Moreover, the observed increase in *Alistipes* abundance following Ap77 treatment may further contribute to its anti-inflammatory and metabolic benefits, as members of this genus have been associated with improved gut barrier function and reduced hepatic inflammation [13,50]. With the advancement of microbiome profiling techniques such as 16S rRNA sequencing, gut microbiota composition and function are increasingly recognized as promising biomarkers for disease stratification and therapeutic response monitoring in chronic metabolic disorders. These tools may support the development of personalized microbial-targeted interventions for MASLD in the future [61].

Despite the promising findings, several limitations should be acknowledged. First, this study was conducted in an animal model, and the applicability of the results to human MASLD patients requires validation. Second, due to the limited resolution of 16S rRNA sequencing, microbial shifts were assessed only at the genus level, restricting species-level interpretation. Lastly, the causal mechanisms underlying the observed microbial and metabolic changes remain to be fully elucidated and warrant further investigation through targeted functional assays and fecal microbiota transplantation experiments.

## 5. Conclusions

**Figure 8:**
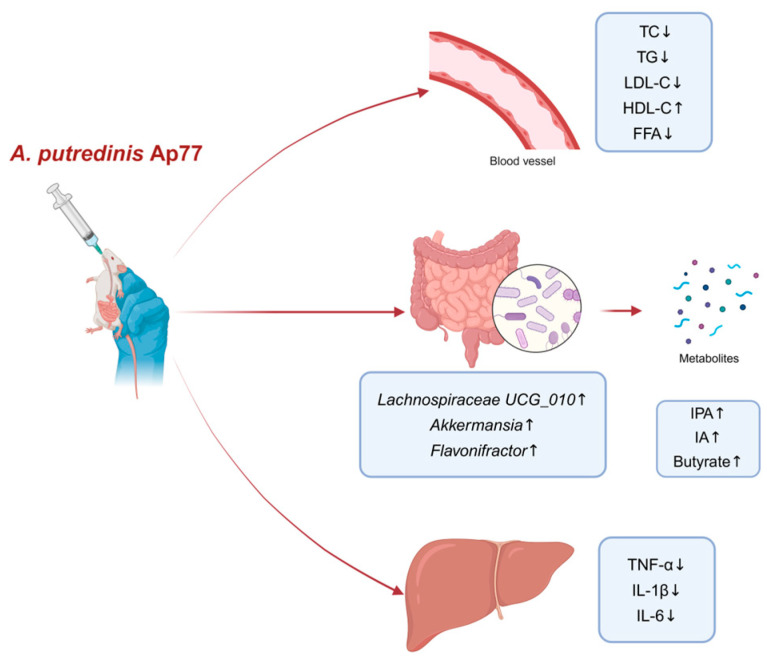
Mechanisms by *A. putredinis* Ap77 alleviates MASLD in HFD-fed rats. *A. putredinis* Ap77 improves lipid metabolism, reshapes the gut microbiota, promotes the production of beneficial microbial metabolites (IPA, IA, butyrate), and reduces hepatic inflammation.

Our study demonstrates that *A. putredinis* strain Ap77 alleviates hepatic lipid accumulation, inflammation, and gut microbiota dysbiosis in rats with HFD-induced MASLD. These improvements are associated with the modulation of inflammation-related pathways, the enrichment of beneficial gut genera, and elevated serum levels of IPA, IA, and butyrate, suggesting that Ap77 may help ameliorate metabolic and inflammatory disturbances associated with MASLD (Figure 8). Further studies are warranted to elucidate the precise mechanisms by which Ap77 modulates host metabolism and inflammation.

## Data Availability

All sequencing data are publicly available in the NCBI SRA database (BioProject numbers: PRJNA1253867 and PRJNA1250185).

## Supplementary Materials

The following supporting information can be downloaded at https://www.mdpi.com/article/10.3390/nu17122013/s1.

## Abbreviations

MASLD	Metabolic-dysfunction-associated steatotic liver disease
NAS	NAFLD Activity Score
NC	Normal chow diet
HFD	High-fat diet
ALT	Alanine aminotransferase
AST	Aspartate aminotransferase
ALP	Alkaline phosphatase
TC	Total cholesterol
TG	Triglyceride
LDL-C	Low-density-lipoprotein cholesterol
HDL-C	High-density-lipoprotein cholesterol
FFA	Free fatty acid
DEGs	Differentially expressed genes
KEGG	Kyoto Encyclopedia of Genes and Genomes
GSEA	Gene set enrichment analysis
TNF-α	Tumor necrosis factor alpha
IL-1β	Interleukin-1 beta
IL-6	Interleukin-6
ASV	Amplicon Sequence Variant
PCoA	Principal Coordinate Analysis
LEfSe	Linear Discriminant Analysis Effect Size
SCFAs	Short-chain fatty acids
IPA	Indole-3-propionic acid
IA	Indoleacrylic acid
