# Supplementary material for: Alistipes putredinis Ameliorates Metabolic Dysfunction-Associated Steatotic Liver Disease in Rats via Gut Microbiota Remodeling and Inflammatory Suppression
Source: Nutrients. 2025 Jun 16;17(12):2013. doi: 10.3390/nu17122013 (PMC12196099; doi:10.3390/nu17122013)
Supplement: Supplementary file 1 [file nutrients-17-02013-s001.zip › nutrients-3702816-supplementary.pdf]

Supplementary figures

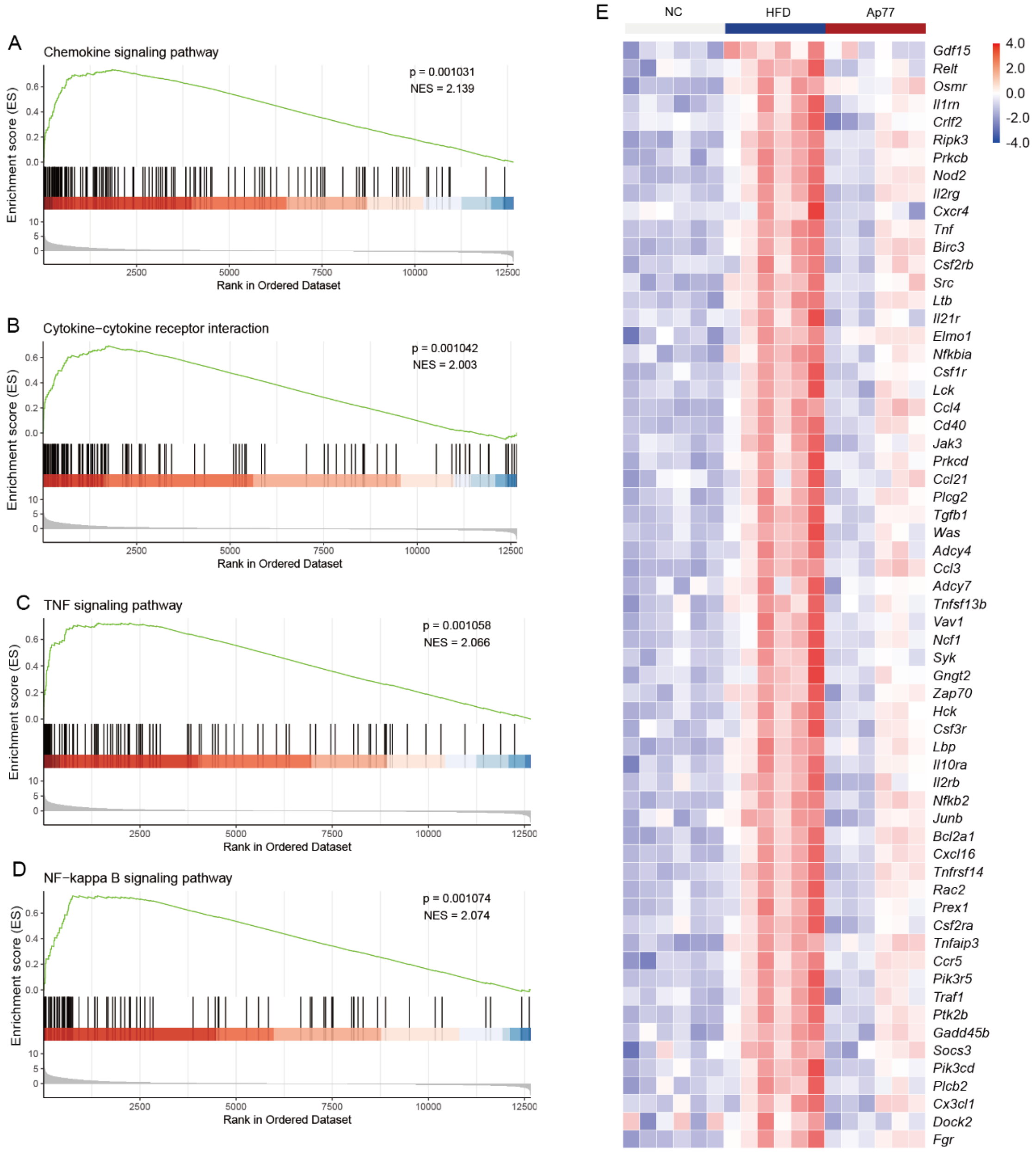

Figure S1. Gene expression changes in inflammation-related pathways. (A-D) GSEA plots showing enrichment of inflammation-related pathways in the HFD group compared to NC, including chemokine signaling (A), cytokine–cytokine receptor interaction (B), TNF signaling (C), and NF-κB signaling pathways (D). **(E)** Heatmap showing DEGs involved in these pathways across NC, HFD, and Ap77 groups. Red indicates higher expression and blue indicates lower expression.

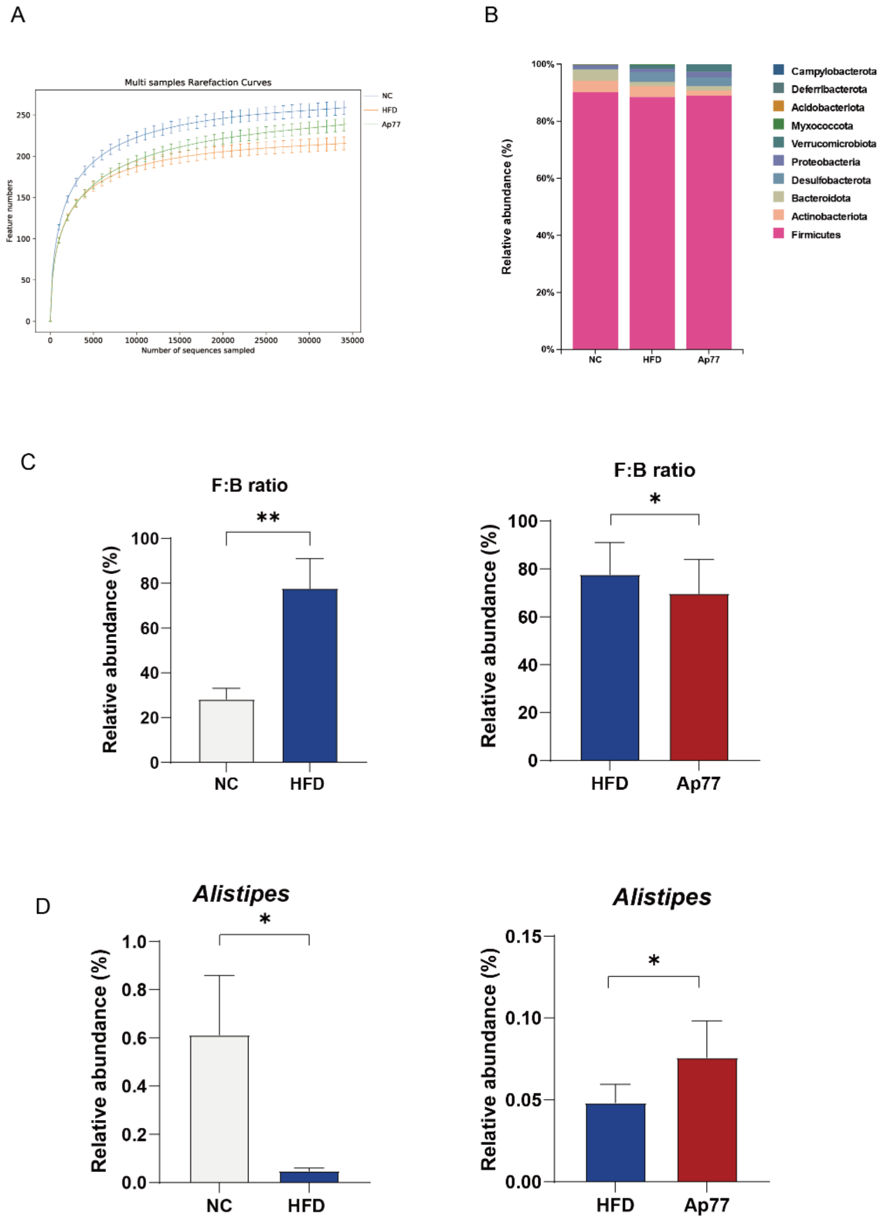

Figure S2. Effects of Ap77 on gut microbiota. (A) Rarefaction curves showing species richness as a function of sequencing depth for each group. **(B)** Phylum level composition of fecal microbiota. **(C)** Firmicutes to Bacteroidota (F:B) ratio. **(D)** Relative abundance of the *Alistipes* genus. Data is presented as mean ± SD (n = 8 per group). * $P < 0.05$, ** $P < 0.01$.

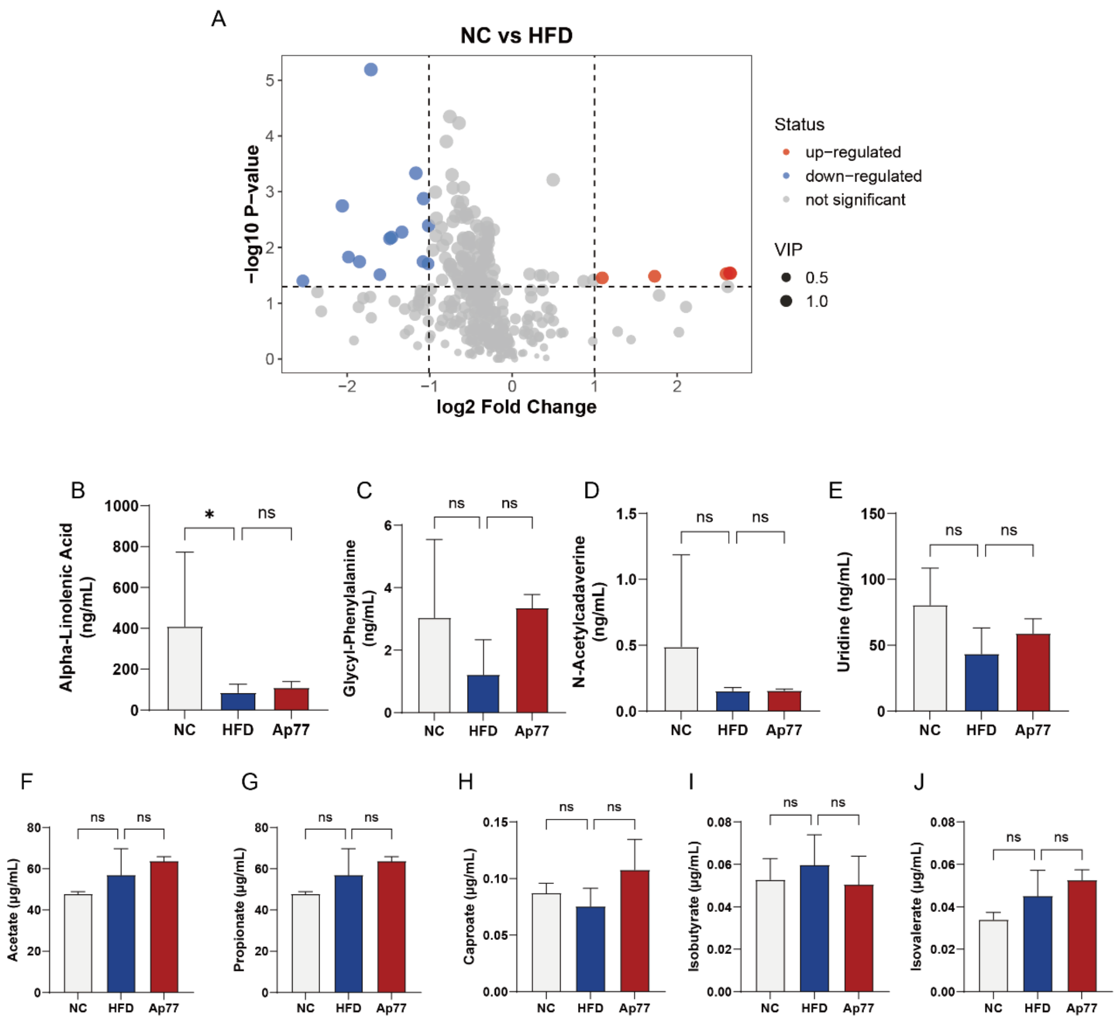

Figure S3. Serum metabolite. (A) Volcano plot of differential metabolites between NC and HFD groups. Red and blue dots represent significantly upregulated and downregulated metabolites, respectively; dot size indicates VIP score. (B-E) Serum levels of α-linolenic acid (ALA), glycyl-phenylalanine (Gly-Phe), N-acetylcadaverine, and uridine. (F-J) Serum concentrations of SCFAs, including acetate, propionate, caproate, isobutyrate, and isovalerate. Data are presented as mean ± SD (n = 4 per group). * $P < 0.05$, ns, not significant.
